# Construction and Evaluation of Quadruple-Gene-Deleted Pseudorabies Virus Platforms for ASFV Antigen Delivery

**DOI:** 10.1155/tbed/3628600

**Published:** 2025-07-16

**Authors:** Hui Li, Ruhai Guo, Yanqing Jia, Xiao Zhang, Zishan Liu, WenLi Shi, Ruochen Hu, YiNing Zhang, Saba Nasir, Likang Han, Xinxin Qiu, Xinglong Wang

**Affiliations:** ^1^College of Veterinary Medicine, Northwest A&F University, Yangling, Shanxi, China; ^2^Department of Animal Engineering/Engineering Research Center of Animal Disease Prevention and Control, Universities of Shaanxi Province, Yangling Vocational and Technical College, YangLing, Shaanxi Province, China; ^3^College of Veterinary Medicine, Gansu Agricultural University, Anning, Gansu, China

**Keywords:** African swine fever virus, antigen delivery, pseudorabies virus platforms, recombinant virus, vaccine

## Abstract

African Swine Fever (ASF) is a highly lethal viral disease in swine. The emergence and rapid spread of African Swine Fever virus (ASFV) in China, since 2018 have caused significant economic losses to the pig farming industry. The complexity of ASFV has impeded the development of effective vaccines, and with no commercial vaccines currently available in China, highlighting the urgent need for safe and efficacious vaccine candidates. In this study, we utilized a highly immunogenic quadruple-gene-deleted recombinant pseudorabies virus (PRV) strain (rPRV SX-10ΔUL24/TK/gI/gE) as a vector to construct two recombinant viral strains expressing ASFV p54, p72, CD2v, and pp62 proteins using the HDR-CRISPR/Cas9 system. These strains, rPRV-p54+p72 and rPRV-CD2v+pp62, demonstrated stable genetic characteristics and efficiently expressed and delivered heterologous proteins while maintaining biological properties similar to their parental strain. Safety evaluation revealed that both recombinant strains exhibited favorable safety profiles in immunized mice and piglets. Furthermore, the strains induced robust humoral and cellular immune responses, as evidenced by specific antibody enzyme-linked immunosorbent assay (ELISA), lymphocyte proliferation assays, and analysis of CD3+, CD4+, and CD8+ T lymphocytes. These findings suggest that rPRV-p54+p72 and rPRV-CD2v+pp62 are promising bivalent vaccine candidates for protecting against both PRV and ASFV infections.

## 1. Introduction

African Swine Fever (ASF) is a highly contagious viral disease caused by African Swine Fever virus (ASFV) [1, 2]. The disease primarily affects domestic and wild pigs, characterized by high morbidity and mortality rates. Clinical manifestations include acute hemorrhagic fever, persistent skin ulceration and arthritis, with infected animals typically succumbing to death within 4–10 days postinfection [3–5]. Due to its significant impact, ASF is designated as a notifiable disease by the World Organisation for Animal Health (WOAH) [6]. Since its first description in Kenya in 1921, the virus has exhibited extensive geographical spread, initially to neighboring countries, and subsequently emerging in the Caucasus region, Georgia, Russia, and various European countries during 2007–2017 [7, 8]. By 2018, ASFV had expanded its reach to China, Mongolia, and most regions of Southeast Asia and Oceania [3, 9]. This widespread outbreak has precipitated severe socioeconomic consequences, posing substantial threats to the global swine industry, food security, international trade, and market stability [10, 11].

ASFV is a large, enveloped, structurally complex double-stranded DNA arbovirus with a genome size of ~170–194 kb. The viral genome encodes 150–200 proteins, including 68 structural proteins and over 100 nonstructural proteins, with approximately half of its genes having no known or predicted functions [12–16]. The complexity of the ASFV genome, combined with the lack of heterologous protection between strains and the virus's sophisticated immune evasion mechanisms, has significantly hindered vaccine development, resulting in the current absence of effective commercial vaccines or therapeutic drugs [17, 18]. Current control strategies are limited to surveillance, culling of infected and exposed animals, and implementation of strict biosecurity measures to contain viral spread. Consequently, the development of effective vaccines against ASFV remains a critical global priority for the prevention and control of ASF.

Over the past decades, researchers have pursued various strategies to develop safe and effective vaccines against ASFV, including inactivated vaccines, attenuated vaccines, subunit vaccines, and viral vector vaccines. Traditional inactivated vaccines have consistently failed to provide protection against ASFV infection [19, 20]. While modified live virus (MLV) vaccines based on virulence-associated gene deletions have demonstrated protective efficacy against virulent strains [21–27], substantial safety concerns remain regarding vaccine virus persistence, potential virulence reversion, and possible recombination with wild-type strains in swine populations [28]. These limitations have led to increased interest in viral vector vaccines as a safer alternative approach. Studies utilizing combinations of one or several antigens have shown promising results, including delayed onset of viremia, extended survival times, and induction of specific antibodies, although complete protection remains elusive [29, 30]. Additionally, investigations of antigen combinations from various ASFV genes delivered through adenovirus, pseudorabies virus (PRV), alphavirus, and vaccinia virus vectors have demonstrated the ability to elicit robust antigen-specific cellular responses [31–34]. These findings suggest that vector-based vaccines may represent a promising strategy for ASF prevention.

PRV is an ideal vector for vaccine development due to its large genome and the ability to delete nonessential genes (TK, gI, gE, and PK) without compromising replication or growth. It efficiently expresses protective antigens from various pathogens, including viruses, bacteria, and parasites, making it suitable for multivalent vaccine development [35–38]. Furthermore, extensive studies have shown that ASFV antigens, including CD2v, p72, p54, p30, and pp62, expressed through viral vectors, elicit robust humoral and cellular immune responses with protective effects [39–42]. These findings suggest that the strategic selection of multiple protective antigens combined with effective delivery systems capable of inducing protective immunity may be critical for successful ASFV vaccine development.

In this study, we used a previously constructed quadruple-gene-deleted recombinant PRV strain with demonstrated immunogenicity as a vector. Through Clustered Regularly Interspaced Short Palindromic Repeats (CRISPR)/Cas9 technology and homologous recombination, we inserted ASFV genes encoding p54, p72, CD2v, and pp62 into the recombinant PRV strain, generating rPRV-p54+p72 and rPRV-CD2v+pp62. We comprehensively evaluated their safety profiles and ability to induce humoral and cellular immune responses in mice and piglets, aiming to develop a bivalent vaccine candidate against both ASF and pseudorabies.

## 2. Materials and Methods

### 2.1. Cells, Viruses, and Animals

BHK-21, PK-15, and HEK 293T cell lines were obtained from the American Type Culture Collection (ATCC). Cells were maintained in Dulbecco's Modified Eagle's Medium (DMEM) supplemented with 10% fetal bovine serum (FBS; Gibco), 100 U/mL penicillin, and 100 mg/mL streptomycin at 37°C in a humidified atmosphere containing 5% CO_2_. The PRV SX-10 strain was previously isolated in our laboratory in 2015. Using PRV SX-10 as the parental strain, we constructed the recombinant virus rPRV ΔTK/UL24/gI/gE, whose characteristics have been previously described [43]. Six-week-old specific-pathogen-free (SPF) female Kunming mice were purchased from Chengdu Dossy Experimental Animals Co., Ltd. (Chengdu, China). Four-week-old American Landrace piglets (7–9 kg) were obtained from Shaanxi Shunxin Breeding Pig Co., Ltd. (Shaanxi, China). Prior to experimentation, all piglets were confirmed to be serologically negative for porcine reproductive and respiratory syndrome virus (PRRSV), porcine circovirus 2 (PCV2), classical swine fever virus (CSFV), porcine parvovirus (PPV), and PRV using enzyme-linked immunosorbent assay (ELISA).

### 2.2. Construction of Recombinant PRV Strains

Two recombinant PRV strains expressing ASFV proteins were constructed: rPRV-p54+p72 (expressing p54 and p72) and rPRV-CD2v+pp62 (expressing CD2v and pp62). The gene sequences of ASFV p54, p72, CD2v, and pp62 were codon-optimized and fuzed with Flag and HA tags to generate synthetic fragments p54-Flag, p72-HA, CD2v-Flag, and pp62-HA. The quadruple-gene-deleted PRV strain (ΔUL24/TK and ΔgE/gI) served as the parental strain. CMV promoter-driven p54-Flag and CD2v -Flag were inserted into the UL24/TK deletion site, while CMV promoter-driven p72-HA and pp62-HA were inserted into the gE/gI deletion site using homologous recombination (Table 1). This process generated four plasmids: pUC19-TKLA-CMV-p54-Flag-EGFP-polyA-TKRA, pUC19-TKLA-CMV-CD2v-Flag-EGFP-polyA-TKRA, pUC19-gILA-CMV-p72-HA-EGFP-polyA-gERA, and pUC19-gILA-CMV-pp62-HA-EGFP-polyA-gERA.

Single guide RNAs (sgRNAs) targeting TK/UL24 (sgRNA1/2) and gE/gI (sgRNA3/4) were designed using the CRISPR design tool (https://zlab.bio/guide-design-resources) (Table 1). HEK 293T cells were grown to 90% confluency and transfected with the constructed plasmids and sgRNAs. At 20 h posttransfection, cells were infected with rPRV ΔTK/UL24/gI/gE at a multiplicity of infection (MOI) of 0.1. Viral cultures were harvested when 90% of cells showed cytopathic effects (CPE). The recombinant viruses were amplified and purified in BHK-21 cells, and the presence of inserted genes was verified by PCR using specific primers for p54, p72, CD2v, and pp62 (Table 1). Finally, the recombinant viruses were propagated, titrated according to the Reed-Muench method to calculate TCID_50_ values, and stored at −80°C.

### 2.3. Biological Characterization of Recombinant Virus Strains

For plaque size determination, PK-15 cells were grown to 90% confluency in 12-well plates and infected with serial dilutions (10^−2^ – 10^−7^) of rPRV-p54+p72, rPRV-CD2v+pp62, or PRV SX-10 (300 μL/well). Following viral adsorption at 37°C for 1 h, the inoculum was removed and cells were overlaid with 1% methylcellulose. Cells were fixed with 4% paraformaldehyde for 15 min and stained with 0.1% crystal violet for 20 min at 48–60 h postinfection (hpi). Plaque sizes were measured using ImageJ software.

To analyze single-step growth kinetics, PK-15 cells in 12-well plates were infected with rPRV-p54+p72, rPRV-CD2v+pp62, or parental PRV SX-10 at a MOI of 0.01. Viral cultures were harvested at 0, 12, 24, 36, 48, 60, and 72 hpi. The samples were serially diluted (10^−1^ – 10^−10^) and inoculated onto PK-15 cell monolayers in 96-well plates. After observation of CPE, viral titers were determined according to the Reed-Muench method to calculate TCID_50_ values, and stored at −80°C.

The genetic stability of rPRV-p54+p72 and rPRV-CD2v+pp62 was evaluated through 20 serial passages in PK-15 cells (MOI = 0.1). Viral genomic DNA was extracted at passages F5, F10, and F20. PCR analysis was performed to confirm both the deletion of UL24/TK and gE/gI genes and the stable insertion of ASFV p54, p72, CD2v, and pp62 genes (Table 1). PRV SX-10 genomic DNA and sterile double-distilled water were used as positive and negative controls, respectively.

### 2.4. Immunocytochemistry Analysis

Expression of recombinant viral proteins was evaluated by immunocytochemistry. BHK-21 cell monolayers were infected with rPRV-p54+p72 or rPRV-CD2v+pp62. At 24 hpi, cells were fixed with 4% paraformaldehyde for 30 min, permeabilized with phosphate-buffered saline (PBS) containing 0.5% Triton X-100 at 37°C for 10 min, and blocked with 1% bovine serum albumin (BSA) at 37°C for 2 h. Cells were then incubated with mouse anti-Flag monoclonal antibody (Proteintech) and rabbit anti-HA monoclonal antibody (Cell Signaling Technology) for 90 min. After washing with PBS, cells were incubated for 1 h with horseradish peroxidase (HRP)-conjugated goat anti-mouse IgG or goat anti-rabbit IgG (Proteintech) at a 1:5000 dilution. Following PBS washes, cells were stained with 3,3′-diaminobenzidine tetrahydrochloride (DAB) and visualized using an upright microscope.

### 2.5. Immunofluorescence Analysis

HEK 293T cells were grown to 90% confluency in 12-well plates and transfected with the plasmids pUC19-TKLA-CMV-p54-Flag-EGFP-polyA-TKRA, pUC19-TKLA-CMV-CD2v-Flag-EGFP-polyA-TKRA, pUC19-gILA-CMV-p72-HA-EGFP-polyA-gERA, and pUC19-gILA-CMV-pp62-HA-EGFP-polyA-gERA using Turbofect Transfection Reagent (Thermo Fisher Scientific). At 24 h posttransfection, cells were fixed with 4% paraformaldehyde (1 mL/well) and permeabilized with 0.1% Triton X-100 (400 μL/well). After blocking with 1% BSA at 37°C for 2 h, cells were sequentially incubated with mouse anti-Flag monoclonal antibody (1:1000 dilution; Proteintech) and rabbit anti-HA monoclonal antibody (1:1000 dilution; Cell Signaling Technology), followed by Alexa Fluor 594-conjugated goat anti-mouse IgG and Alexa Fluor 488-conjugated goat anti-rabbit IgG (1:1000 dilution; Proteintech). Cell nuclei were counterstained with 4′, 6-diamidino-2-phenylindole (DAPI; Solarbio). Images were acquired using a Leica TCS Sp8 confocal laser scanning microscope.

### 2.6. Western Blot Analysis

Protein expression in recombinant viruses was further evaluated by western blot analysis. HEK-293 cell monolayers were infected with rPRV-p54+p72 or rPRV-CD2v+pp62. At 24 hpi, cells were harvested and lysed in radio immunoprecipitation assay (RIPA) buffer (Solarbio). Total proteins were separated by sodium dodecyl sulfate-polyacrylamide gel electrophoresis (SDS-PAGE) and transferred to polyvinylidene difluoride (PVDF) membranes. After blocking with 5% nonfat milk in phosphate buffered saline with tween (PBST) at room temperature for 2 h and washing three times with PBST, membranes were incubated overnight at 4°C with mouse anti-Flag monoclonal antibody (1:1000 dilution) and rabbit anti-HA monoclonal antibody (1:1000 dilution) diluted in PBST. The membranes were then incubated for 1 h with HRP-conjugated goat anti-mouse IgG or goat anti-rabbit IgG at a 1:5000 dilution. Immunoreactive Sbands were visualized using enhanced chemiluminescence (ECL) substrate (DiNing) and imaged with a chemiluminescence imaging system.

### 2.7. Mouse Immunization and Challenge Study

**Figure 1:**
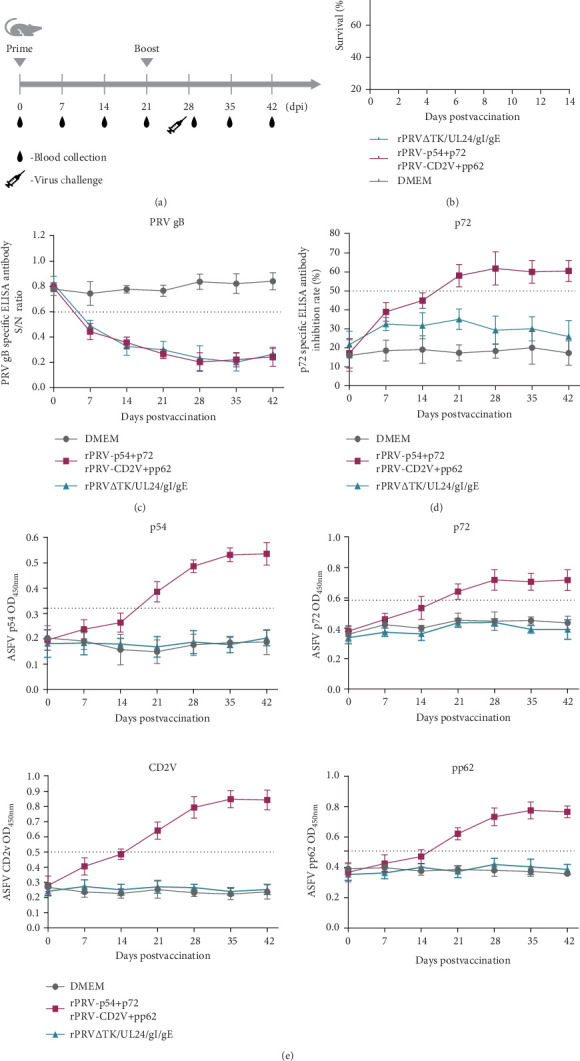
Immunogenicity of rPRV-p54+p72 and rPRV-CD2v+pp62 in mice. (A) Schematic representation of the immunization protocol. Mice were immunized with rPRV-p54+p72, rPRV-CD2v+pp62, or DMEM at indicated time points. (B) Survival rates of mice after immunization with rPRV-p54+p72 and rPRV-CD2v+pp62. (C) PRVgB specific antibody levels in sera of immunized mice detected by ELISA. (D) p72-specific antibody levels in sera of immunized mice detected by commercial blocking ELISA. (E) Specific antibody levels against p54, p72, CD2v, and pp62 in sera of immunized mice detected by established ELISA methods. Serum samples were collected at different time points post-immunization.

Six-week-old female Kunming mice were randomly divided into four groups (*n* = 10 per group), and the immunization schedule is shown in Figure 1A. Mice were initially immunized intramuscularly in the hind leg with viral suspensions (1 × 10^6^ TCID_50_/mL) of rPRV-p54+p72, rPRV-CD2v+pp62, rPRV ΔTK/UL24/gI/gE, or PRV SX-10, while the control group received DMEM only. A booster immunization was administered 14 days after the initial immunization, following the same protocol. All groups were housed separately under standard laboratory conditions with ad libitum access to food and water. Clinical signs and survival rates were monitored daily.

At 28 dpi, surviving mice were challenged intramuscularly with PRV SX-10 (1 × 10^6^ TCID_50_/mL) and monitored for an additional 14 days. Brain, liver, lung, and spleen tissues were collected for histopathological analysis and cell proliferation assays. Blood samples were collected from the retro-orbital sinus at 0, 7, 14, 21, 28, 35, and 42 dpi. Sera were separated by centrifugation (3000 × *g*, 10 min, 4°C) and stored at −80°C until analysis.

### 2.8. Piglet Immunization Study

**Figure 2:**
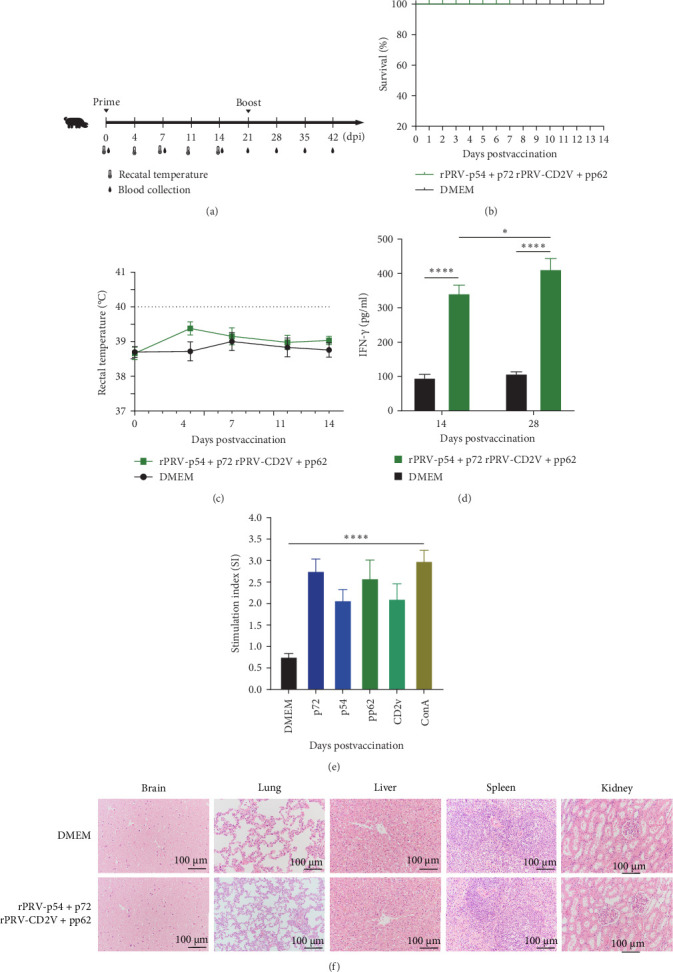
Evaluation of immunogenicity and safety of rPRV-p54+p72 and rPRV-CD2v+pp62 immunization in piglets. (A) Schematic diagram of the prime-boost vaccination schedule in pigs. (B) Survival rate of pigs. (C) Body temperature curve of piglets. (D) Changes in serum IFN-γ levels in piglets after immunization with recombinant virus strains. (E) Effects of p54, p72, CD2v, and pp62 antigen stimulation on PBMCs proliferation in piglets. (F) No histopathological lesions observed in various organs of piglets following immunization with rPRV-p54+p72 and rPRV-CD2v+pp62. H&E, hematoxylin and eosin.

Four-week-old weaned piglets (*n* = 15) were used, and the immunization schedule is shown in Figure 2A. The experimental group (*n* = 10) received intramuscular injections of rPRV-p54+p72 and rPRV-CD2v+pp62 viral suspensions (3 mL, 10^6^ TCID_50_/mL), while the control group (*n* = 5) received 3 mL DMEM only. Animals were housed individually under standard conditions with ad libitum access to food and water. Clinical signs and survival rates were monitored daily.

A booster immunization was administered at 21 dpi following the same protocol. Brain, lung, spleen, kidney, and liver tissues were collected for histopathological analysis. Additionally, peripheral blood mononuclear cells (PBMCs) were isolated for cell proliferation assays. Blood samples (5 mL) were collected from the anterior vena cava at 0, 7, 14, 21, 28, 35, and 42 dpi. Sera were separated by centrifugation (3000 × *g*, 10 min, 4°C) and stored at −80°C until analysis.

### 2.9. ELISA

PRVgB-specific antibody titers in mouse and piglet sera were evaluated using a PRVgB Antibody ELISA test kit (JNT) according to the manufacturer's instructions. The results were calculated as S/N = sample optical density (OD) OD_450_/negative control (NC) OD OD_450_, with S/N ≤ 0.6 considered positive and S/N > 0.6 is considered to be negative.

ASFV p54-specific antibody titers in piglet sera were measured using an ASFV ELISA antibody test kit (p54) following the manufacturer's protocol. The results were calculated as S/P = (sample OD OD_450_ − mean NC OD OD_450_)/(mean positive control OD OD_450_ − mean NC OD OD_450_), with S/P ≥ 0.45 considered positive and S/P < 0.45 negative.

ASFV p72-specific antibody titers in mouse and piglet sera were evaluated using an ASFV blocking ELISA antibody detection kit (p72) according to the manufacturer's guidelines. The results were calculated as blocking rate (%) = (mean NC OD OD_450_ − sample OD OD_450_)/mean NC OD OD_450_ × 100%. The samples with blocking rates ≤ 40% were considered negative, ≥50% positive, and between 40% and 50% suspicious.

Due to limited commercial availability of ASFV CD2v and pp62 test kits and the need for viral antigens in cell proliferation assays, recombinant ASFV proteins were expressed. Briefly, PCR products of ASFV p54, p72, CD2v, or pp62 genes were cloned into pET-30 or pCold-SUMO vectors (Figure S1A) and transformed into *Escherichia coli* BL21. IPTG induction yielded soluble p54 and pp62 proteins, and inclusion body forms of p72 and CD2v proteins (Figure S1B). The recombinant proteins were purified by Ni^2+^ affinity chromatography and verified by SDS-PAGE (Figure S1C). Western blot analysis using anti-His antibody (1:1000) showed strong reactivity (Figure S1D). Antigen and antibody concentrations were optimized by checkerboard titration (P/N values) (Figure S1E). The cut-off values (p54 OD_450_ ≥ 0.321, p72 OD_450_ ≥ 0.58, CD2v OD_450_ ≥ 0.5, pp62 OD_450_ ≥ 0.5) were determined as the mean negative sera OD_450_ plus three standard deviations. An indirect ELISA method was established using the purified recombinant proteins as antigens.

Serum cytokine levels in mice (IL-12, IL-4, IL-6, IL-10, IFN-γ, and TNF-α) were measured using commercial ELISA test kits (catalog numbers KE10004, KE10010, KE10007, KE10103, KE10094, and KE10002, Proteintech). Porcine IFN-γ levels were assessed using an ELISA test kit (catalog number EPC004g, Neobioscience). All assays were performed strictly according to manufacturers' instructions, with cytokine concentrations determined from independently constructed standard curves for each assay.

### 2.10. Lymphocyte Proliferation Assay

The lymphocyte proliferation assay was performed to evaluate the proliferative capacity of mouse splenic lymphocytes and piglet PBMCs in response to specific protein stimulation. The preparation procedures for mouse splenic lymphocytes and piglet PBMCs were as follows:

For mouse splenic lymphocytes isolation at 28 dpi, the spleen was mechanically homogenized in PBS using the plunger end of a syringe. The homogenate was filtered through a cell strainer into a collection tube. Following erythrocyte lysis and PBS washing, cells were enumerated and resuspended in RPMI 1640 medium supplemented with 10% FBS. For the proliferation assay, splenic cells were seeded into 96-well plates at a density of 3 × 10^6^ cells/mL (100 μL/well).

For piglet PBMC isolation at 28 dpi, PBMCs were isolated from blood samples using a lymphocyte separation kit (Solarbio) according to the manufacturer's instructions. Briefly, anticoagulated whole blood was diluted and carefully layered onto an equal volume of peripheral blood mononuclear cell separation solution. Following centrifugation, the cloudy PBMC layer was collected, subjected to erythrocyte lysis, and washed with PBS. Cells were then resuspended in RPMI 1640 containing 10% FBS at 2 × 10^6^ cells/mL and seeded into 96-well plates (100 μL/well).

Subsequently, cells were stimulated with recombinant ASFV proteins (p54, p72, CD2v, or pp62). Concanavalin A, unstimulated cells, and RPMI-1640 medium served as positive, negative, and blank controls, respectively. After 72 h of incubation, 10 μL of Cell Counting Kit-8 (CCK-8) reagent was added to each well, followed by 2 h of incubation. The OD was measured at 450 nm using a microplate reader. The stimulation index (SI) was calculated using the following formula: SI = (ODexperimental − ODblank)/(ODnegative control − ODblank).

### 2.11. Flow Cytometric Analysis of CD3/CD4/CD8 Mouse Splenic Lymphocytes

Mouse splenic cells were isolated at 28 dpi and adjusted to a concentration of 1 × 10^7^ cells/mL. The cells were incubated with the following fluorochrome-conjugated monoclonal antibodies (Proteintech): CoraLite Plus 488-conjugated anti-mouse CD3 (clone 17A2), PE-conjugated anti-mouse CD4 (clone RM4-5), and APC-conjugated anti-mouse CD8a (clone 53–6.7). Flow cytometry was performed to determine the proportions of CD4+ T cells, CD8+ T cells, and CD3+ T cells within splenic T lymphocyte subpopulations. The percentages of CD3+, CD4+, and CD8+ T cells were analyzed using a flow cytometer and FlowJo software (version 10.8.1).

### 2.12. Histopathological Examination

For histopathological examination, brain, lung, and splenic tissues were collected from euthanized mice and piglets. The tissues were fixed in 4% paraformaldehyde and subsequently submitted to Y&K Biosciences (Shanxi, Chian) for paraffin embedding, sectioning, and hematoxylin and eosin (H&E) staining. The stained sections were examined under light microscopy for pathological changes.

### 2.13. Statistical Analysis

Statistical differences between groups were analyzed using unpaired *t*-test or two-way analysis of variance (ANOVA) with GraphPad Prism software (version 9.5; GraphPad Software, San Diego, CA, USA). Significant differences between groups are indicated by asterisks: *⁣*^*∗*^*p* < 0.05, *⁣*^*∗∗*^*p* < 0.01, *⁣*^*∗∗∗*^*p* < 0.001. Data are presented as mean ± standard deviation (SD) from at least three independent experiments.

## 3. Results

### 3.1. Construction of Recombinant PRV Strains rPRV-p54+p72 and rPRV-CD2v+pp62

**Figure 3:**
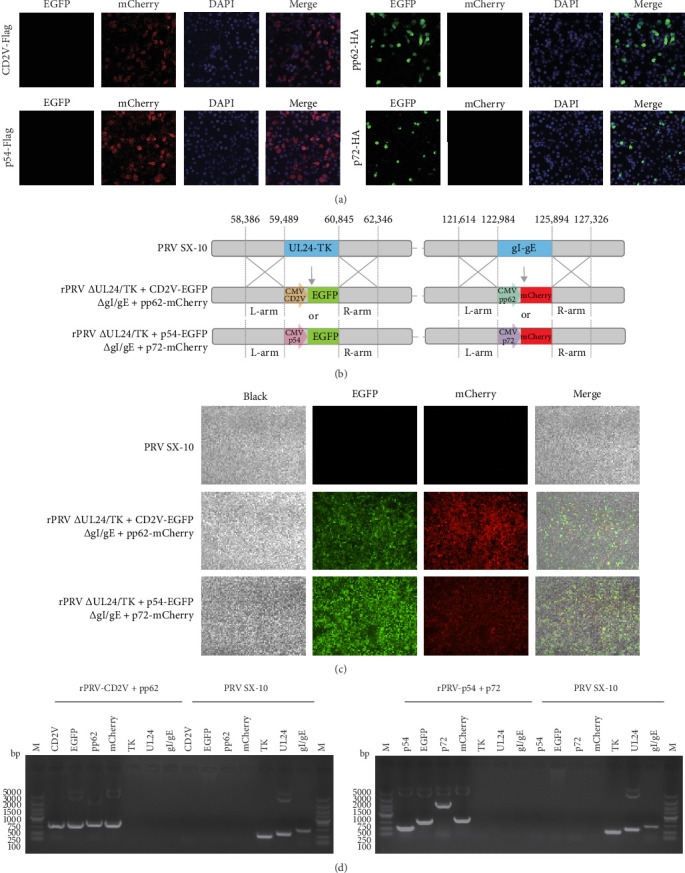
Construction of recombinant virus strains rPRV-p54+p72 and rPRV-CD2v+pp62. (A) Verification of the optimized ASFV p54, p72, CD2v, and pp62 gene expression in BHK-21 cells at 24 h by IFA. (B) and (C) Schematic diagram of the construction and purification of recombinant virus strains rPRV-p54+p72 and rPRV-CD2v+pp62. (D) PCR identification of UL24/TK and gI/gE deletions, along with ASFV p54, p72, CD2v and pp62 insertions in recombinant viruses: rPRV-p54+p72 and rPRV-CD2v+pp62.

To develop a safe and effective ASFV vaccine, we used our previously constructed quadruple-gene-deleted PRV strain as a vector. The ASFV p54, p72, CD2v, and pp62 gene sequences were fuzed with Flag and HA tags, codon-optimized, and synthesized to generate pUC19 constructs and subsequent recombinant virus strains. Expression of ASFV antigens in BHK-21 cells was confirmed by indirect immunofluorescence (Figure 3A). Through CRISPR/Cas9-mediated genome editing and homologous recombination, we inserted CMV promoter-driven ASFV antigens at the deleted UL24/TK and gI/gE loci. Following multiple rounds of plaque purification, we obtained two recombinant PRV strains: rPRV ∆UL24/TK + p54-EGFP/∆gI/gE+p72-mCherry expressing ASFV p54 and p72 proteins, and rPRV ∆UL24/TK + CD2v-EGFP/∆gI/gE+pp62-mCherry expressing ASFV CD2v and pp62 proteins, abbreviated as rPRV-p54+p72 and rPRV-CD2v+pp62, respectively (Figure 3B,C). PCR analysis confirmed the successful deletion of UL24/TK and gE/gI genes and integration of p54, p72, CD2v, and pp62 genes in the recombinant strains (Figure 3D).

### 3.2. Biological Characteristics of Recombinant PRV Strains rPRV-p54+p72 and rPRV-CD2v+pp62

**Figure 4:**
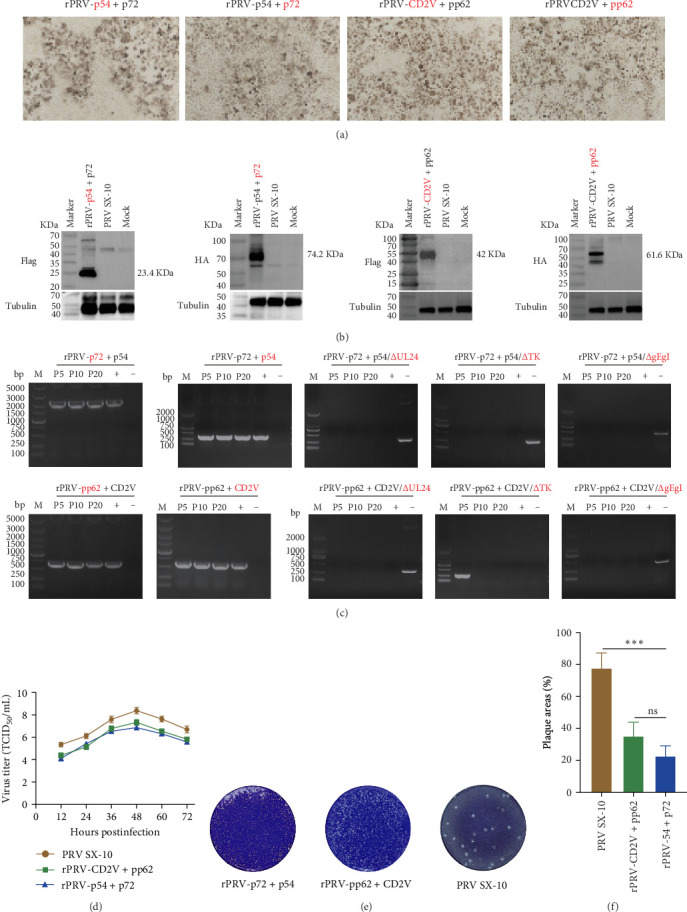
Characterization of rPRV-p54+p72 and rPRV-CD2v+pp62. (A) Assessment of antigen expression in rPRV-ASFV by immunocytochemistry. (B) Analysis of antigen expression in HEK-293T cells infected with rPRV-p54+p72 and rPRV-CD2v+pp62 by western blot. (C) PCR verification of UL24/TK and gI/gE gene deletions and the stable integration of ASFV p54, p72, CD2v and pp62 genes in rPRV-p54+p72 and rPRV-CD2v+pp62 after 20 serial passages. (D) Growth curves of the parental strain PRV SX-10, rPRV-p54+p72 and rPRV-CD2v+pp62. (E) and (F) Measurement of plaque size and morphology of rPRV-p54+p72, rPRV-CD2v+pp62 and PRV SX-10. The plaque areas were counted and calculated using ImageJ software. *⁣*^*∗∗∗*^*p* < 0.001, *ns*: not significant.

To evaluate the capability of recombinant viruses to effectively deliver and mediate transgene expression, BHK-21 cells were infected with rPRV-p54+p72 and rPRV-CD2v+pp62, and subsequently analyzed by Western blotting and immunocytochemistry. Immunocytochemistry revealed robust expression of ASFV antigens p54, p72, CD2v, and pp62 in infected cells (Figure 4A), which was further validated by Western blot analysis (Figure 4B). The genetic stability of rPRV-p54+p72 and rPRV-CD2v+pp62 was assessed through serial passaging for 20 generations, with PCR analysis confirming stable maintenance of p54, p72, CD2v, and pp62 genes (Figure 4C). One-step growth curves revealed that the recombinant strains had growth kinetics similar to the parental strain, PRV SX-10, although with slightly lower viral titers (Figure 4D). Moreover, plaque morphology analysis showed that the recombinant viruses formed smaller plaques compared to the parental strain (Figure 4E).

### 3.3. In Vivo Expression of Recombinant PRV Strains rPRV-p54+p72 and rPRV-CD2v+pp62

**Figure 5:**
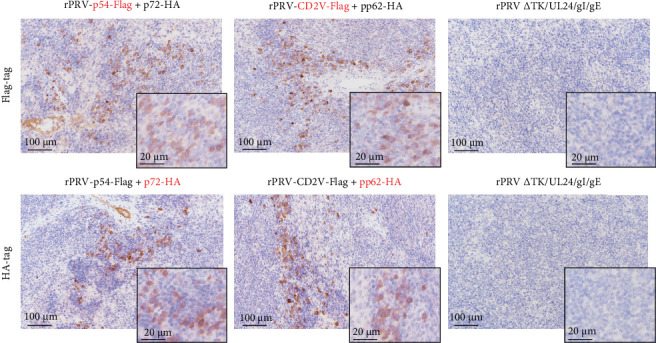
Immunohistochemical detection of epitope-tagged ASFV antigens in mouse spleen. Representative images showing positive immunostaining (brown) of ASFV antigens in spleen tissues from mice infected with recombinant PRVs. Flag antibody was used to detect p54 and CD2v expression, while HA antibody was used to detect p72 and pp62 expression in the recombinant virus strains.

To evaluate the in vivo delivery and expression of exogenous proteins by rPRV-p54+p72 and rPRV-CD2v+pp62, spleens were collected from infected mice 3 days post challenge(dpc)and analyzed by immunohistochemistry using anti-Flag and anti-HA antibodies. Robust expression of p54, p72, CD2v, and pp62 was detected in the spleens of mice immunized with rPRV-p54+p72 and rPRV-CD2v+pp62, whereas no specific staining was observed in mice infected with rPRV ΔTK/UL24/gI/gE (Figure 5).

### 3.4. Recombinant PRV Strains Elicit Specific Humoral and Cellular Immune Responses in Mice

Safety assessment of the recombinant viruses was conducted by immunizing mice with rPRV-p54+p72 and rPRV-CD2v+pp62. Neither pruritus, scratching behavior, nor mortality was observed in mice receiving the recombinant strains, in contrast to those inoculated with PRV SX-10 (Figure 1A,B). Antibody responses against PRVgB, p54, p72, CD2v, and pp62 were evaluated using both commercial and in-house ELISA methods. Mice immunized with rPRV-p54+p72, rPRV-CD2v+pp62, or rPRV ΔTK/UL24/gI/gE developed robust PRVgB-specific antibody responses by 28 dpi, while the DMEM control group showed no response (Figure 1C). Specific antibodies against p54, p72, CD2v, and pp62 were significantly detected in sera from mice immunized with rPRV-p54+p72 and rPRV-CD2v+pp62, compared to rPRV ΔTK/UL24/gI/gE and DMEM groups, as confirmed by both commercial and in-house ELISA methods (Figure 1D,E). These results demonstrate that the recombinant PRV strains successfully induced ASFV-specific humoral immune responses while maintaining their ability to elicit anti-PRVgB antibodies.

**Figure 6:**
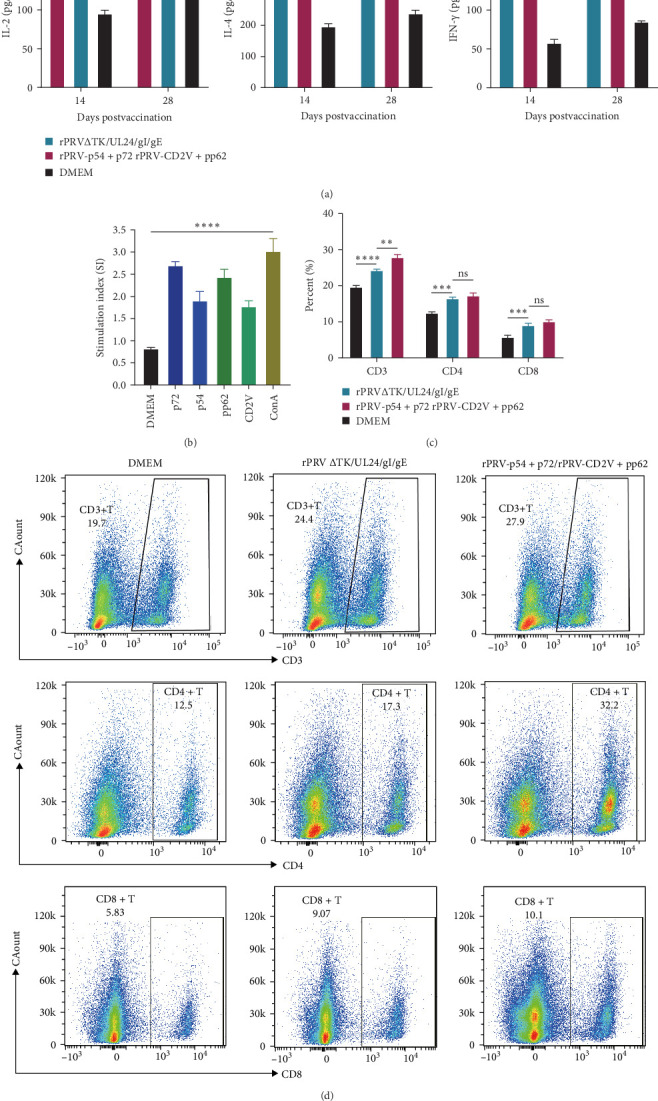
Effects of p54, p72, CD2v, and pp62 antigen immunization on immune responses in mice. (A) Detection of IL-2, IL-4, and IFN-γ levels secreted by splenocytes from mice immunized with rPRV-p54+p72 and rPRV-CD2v+pp62 using ELISA. (B) Effects of p54, p72, CD2v, and pp62 antigen stimulation on mouse splenocyte proliferation. (C, D) Flow cytometric analysis of CD3+, CD4+, and CD8+ T cell percentages in spleens of mice immunized with different antigens. *⁣*^*∗*^*p* < 0.05, *⁣*^*∗∗*^*p* < 0.01, *⁣*^*∗∗∗*^*p* < 0.001. *ns*: not significant.

To assess cellular immune responses, we measured cytokine production, lymphocyte proliferation, and T cell subset distribution. ELISA analysis showed elevated IL-2, IL-4, and IFN-γ levels in mice immunized with rPRV-p54+p72, rPRV-CD2v+pp62, or rPRV ΔTK/UL24/gI/gE compared to DMEM group (Figure 6A). At 28 dpi, splenic lymphocytes from immunized mice showed significant proliferation when stimulated with purified p54, p72, CD2v, and pp62 proteins (Figure 6B). Flow cytometric analysis revealed significantly higher percentages of CD3+, CD4+ and CD8+ T cells in rPRV-p54+p72 and rPRV-CD2v+pp62 group compared to DMEM group, indicating enhanced T cell-mediated immune responses following immunization (Figure 6C,D).

### 3.5. rPRV-p54+p72 and rPRV-CD2v+pp62 Protected Mice against Challenge With PRV Variant Strain

**Figure 7:**
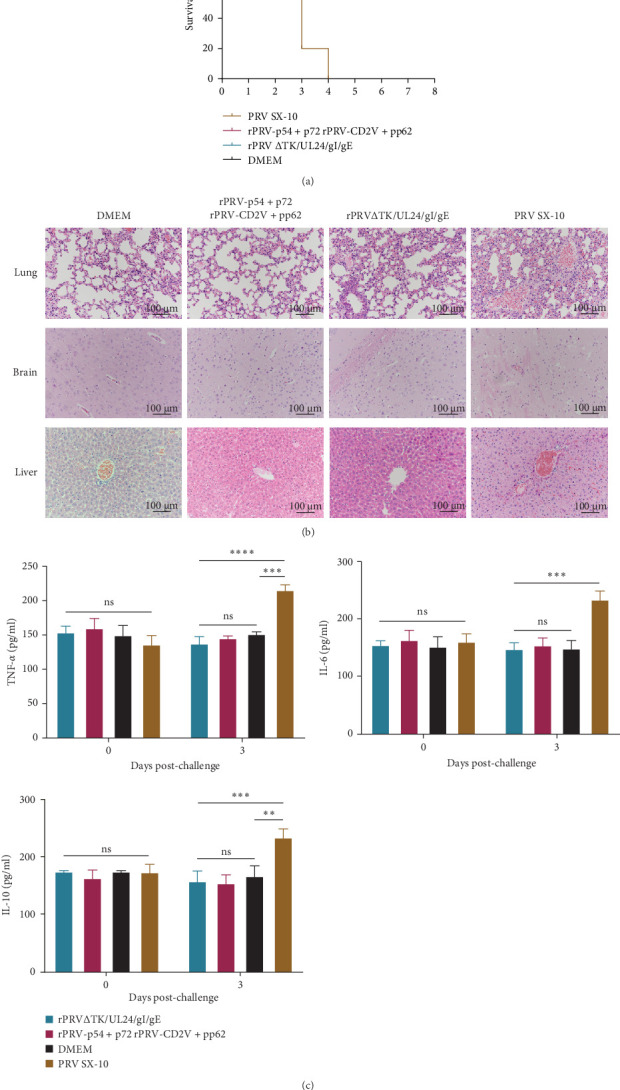
rPRV-p54+p72 and rPRV-CD2v+pp62 protected mice against challenge with PRV SX-10 infection. (A) Survival rates of mice after challenge with PRV SX-10 strain. (B) Histopathological lesions in various organs of mice following PRV challenge. Tissue samples were collected as indicated for histopathological examination. (C) Cytokine expression levels of TNF-α, IL-6, and IL-10 in mouse sera were detected at 3 dpc using ELISA. *⁣*^*∗*^*p* < 0.05, *⁣*^*∗∗*^*p* < 0.01, *⁣*^*∗∗∗*^*p* < 0.001, *⁣*^*∗∗∗∗*^*p* < 0.0001, and *ns* as not significant.

To evaluate protective efficacy, mice were intramuscularly challenged with PRV SX-10 at 28 dpi and monitored for 14 days. Mice in the PRV SX-10 group developed typical symptoms, including pruritus, alopecia, and circling behavior, between 2 and 4 days post-challenge, with 100% mortality. In contrast, mice immunized with rPRV-p54+p72, rPRV-CD2v+pp62, or rPRV ΔTK/UL24/gI/gE remained clinically healthy, achieving 100% survival, similar to the DMEM group (Figure 7A). Histopathological analysis showed no significant tissue changes in the immunized groups, while the PRV SX-10 group exhibited severe pathological changes, including pulmonary hemorrhage, alveolar wall thickening, perivascular cuffing in the brain, and hepatic hemorrhage with hepatocellular necrosis (Figure 7B). Serum ELISA analysis revealed that at 3 dpc, mice infected with PRV SX-10 exhibited significantly elevated levels of IL-6, IL-10, and TNF-α, whereas mice immunized with recombinant virus strains maintained cytokine levels comparable to those of the DMEM group (Figure 7C).

### 3.6. Safety and Cellular Immune Responses Induced by rPRV-p54+p72 and rPRV-CD2v+pp62 in Piglets

The safety profile of the recombinant viral strains was evaluated in piglets. Following immunization with rPRV-p54+p72 and rPRV-CD2v+pp62, piglets showed no adverse clinical signs, such as pruritus, scratching, or mortality (Figure 2A,B). Body temperature remained within normal ranges throughout the 14 dpi period (Figure 2C). Serum IFN-γ levels were significantly higher in both immunization groups compared to DMEM controls (Figure 2D). At 28 dpi, PBMCs from immunized piglets showed robust proliferative responses upon stimulation with purified p54, p72, CD2v, and pp62 proteins, as assessed by CCK-8 assay (Figure 2E). Histopathological examination of lung, brain, liver, spleen, and kidney tissues revealed no pathological alterations in immunized piglets compared to DMEM group (Figure 2F).

### 3.7. Humoral Immune Responses Induced by rPRV-p54+p72 and rPRV-CD2v+pp62 in Piglets

**Figure 8:**
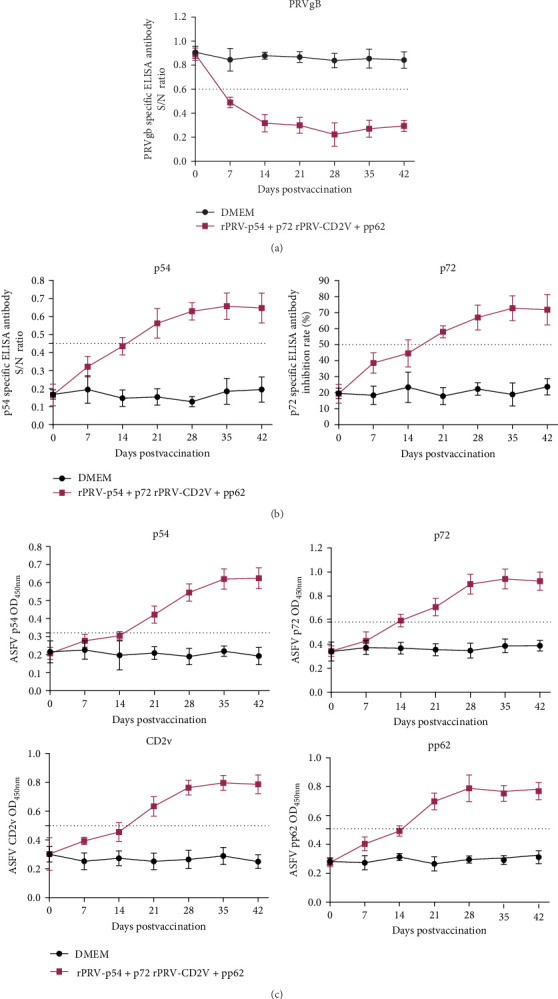
Changes in specific antibody levels in sera of piglets after immunization with rPRV-p54+p72 and rPRV-CD2v+pp62. (A) Anti-PRVgB antibody titers in piglet sera at different time points after immunization. (B) Detection of p54 and p72 antibody titers in piglet sera using commercial ELISA kits. (C) Detection of p54, p72, CD2v, and pp62 antibody titers in piglet sera using in-house developed ELISA methods.

ELISA analysis revealed that both recombinant viral strains induced significantly higher levels of PRVgB antibodies compared to DMEM gruop (Figure 8A). Two weeks after primary immunization, low levels of antibodies against p54, p72, CD2v, and pp62 were detected in sera from rPRV-p54+p72 and rPRV-CD2v+pp62 group. Antibody levels increased substantially following booster immunization in rPRV-p54+p72 and rPRV-CD2v+pp62 group, while no specific antibodies were detected in the DMEM group (Figure 8B,C).

## 4. Discussion

ASFV is a highly pathogenic virus that poses a critical threat to global swine production. The primary transmission route is direct contact between healthy pigs and infected animals or ASFV-contaminated materials, with tick bites from infected soft ticks serving as an additional pathway [5]. Vaccination continues to be recognized as the most cost-effective approach for controlling ASF outbreaks. Previous studies have shown that attenuated vaccines can provide protection against both homologous and heterologous virus challenges, but significant safety concerns have limited their widespread use [44, 45]. Recently, viral vector vaccines have emerged as a safer alternative, offering improved safety profiles and enhanced antigen delivery [34, 46]. In this study, we developed two recombinant PRV vector vaccine candidates using a quadruple gene-deleted strain with enhanced immunogenicity. We evaluated the immunogenic potential of these vaccines in both mouse and piglet models. Our findings demonstrated that both recombinant strains effectively induced robust antibody responses targeting PRVgB and ASFV proteins (p54, p72, CD2v, and pp62), along with significant cellular immune responses.

Several ASFV antigens expressed in viral vectors have demonstrated strong immunogenicity and protective efficacy against ASFV challenge. A cocktail vaccine comprising p54, p72, CD2v, and p30 delivered via Ad2 vector protected pigs against challenge with ASFV China/GD/2019 strain. All 10 vaccinated pigs remained healthy during the 70-day observation period, showing no ASF-related clinical symptoms or viremia [42]. This protection was closely associated with robust humoral and cellular immune responses induced by ASFV antigens. Additionally, various combinations of eight antigens including p54, p72, CD2v, pp62, and p30, delivered using an improved rAd prime and modified vaccinia Ankara (MVA) boost system, protected pigs against lethal disease following challenge with a virulent genotype I ASFV strain [41]. Collectively, these findings demonstrate that viral vector-based vaccines offer a promising approach against ASF, where antigen selection is critical for vaccine efficacy. We engineered two recombinant PRV strains (rPRV-p54+p72 and rPRV-CD2v+pp62) by incorporating ASFV antigens (p54, p72 and CD2v, pp62). Co-immunization with these strains enabled efficient antigen delivery, eliciting robust humoral and cellular immune responses.

Optimal vector selection depends critically on immunogenic potential and sustained foreign protein expression capacity [34]. We employed PRV SX-10, a variant strain isolated in our laboratory in 2015, as the backbone to generate quadruple gene-deleted recombinant vectors expressing ASFV antigens. This strategic approach confers dual protective benefits: mitigating the risk of compromised vaccine efficacy due to PRV variants while simultaneously providing protection against both classical and variant PRV strains alongside ASFV prevention. Furthermore, this vector has demonstrated favorable immunogenicity and protective efficacy against PRV in both mice and piglets, making it an excellent candidate for foreign gene delivery. Our results showed efficient expression of foreign proteins p54, p72, CD2v, and pp62 delivered by the quadruple-gene-deleted PRV vector, as verified through immunocytochemistry and western blot analyses, consistent with previous studies [39]. These results establish a promising platform for future ASFV vaccine development strategies.

Protection against ASFV necessitates both humoral and cellular immune responses, with cellular immunity serving as the predominant mediator. Previous studies have demonstrated that viral vector vaccines expressing ASFV antigens conferred protection against ASFV challenge in the absence of detectable specific antibodies, underscoring the pivotal role of cellular immunity in host protection [29]. IL-2, IL-4, and IFN-γ are key immune mediators that play essential roles in the coordinated action of cellular and humoral immunity [47, 48]. IL-2 primarily promotes T cell proliferation and the formation of immunological memory, ensuring long-term protection against pathogens [49]. IL-4 regulates humoral immune responses by enhancing antibody production [50]. Meanwhile, IFN-γ is a central mediator of cellular immunity, boosting cell-mediated immunity and the macrophage's ability to combat pathogens [51]. The synergistic actions of these cytokines collectively enhance the host's immune defense against viruses, including ASFV. IFN-γ, a key cellular immunity mediator secreted by Th1 cells, was significantly elevated in sera from immunized mice and piglets relative to the DMEM control group. Moreover, enhanced IL-2 and IL-4 secretion in mouse sera demonstrated robust T cell activation and humoral immune responses [48]. The pro-inflammatory cytokines IL-1*β*, IL-6, TNF-α, and MCP-1 function as critical biomarkers of systemic inflammation. Following PRV challenge, the PRV SX-10 group exhibited markedly elevated levels of IL-6, IL-10, and TNF-α compared to the recombinant virus groups, indicating attenuated inflammatory responses and superior protection against PRV variant strains [52, 53]. In lymphocyte proliferation assays, immunized groups exhibited significantly enhanced proliferation rates in response to p54, p72, CD2v, and pp62 protein stimulation relative to the DMEM control group. Helper (CD4+) T cells and CD8+ lymphocyte subsets have been well documented to play vital roles in inducing mucosal antibody responses and immune protection [54, 55]. Notably, our immunological analyses demonstrated significant expansion of CD3+ CD4+ and CD8+ T cell populations in response to immunization with the recombinant strains. Collectively, co-immunization with both recombinant vaccine strains effectively induces robust cellular immune responses.

In conclusion, the engineered recombinant virus strains rPRV-p54+p72 and rPRV-CD2v+pp62 exhibited robust immunogenicity and favorable safety profiles following immunization. This innovative dual-purpose vaccination strategy demonstrates considerable potential for the simultaneous control of PRV and ASFV infections, representing a promising approach for veterinary disease prevention. However, the protective efficacy against viral challenge requires further evaluation.

## Figures and Tables

**Table 1:** Primers used in this study.

Target gene	Primer sequence (5′–3′)
p54-F	ATGGACTCCGAGTTCTTCCA
p54-R	ACAAGGATGACGACGATAAG
p72-F	ATGGCCTCCGGCGGAGCTTT
p72-R	CCGTGCTGCGGTACAGCACC
CD2v-F	ATGATCATCCTGATCTTCCT
CD2v-R	TCCACGTGGACCGGATCATC
pp62-F	ATGCCCTCCAACATGAAGCA
pp62-R	ACGATGTTCCAGATTACGCT
CMV-p54-F	ACGTCGAGCACGCGCATGCGACATTGATTATTGACTAG
CMV-p54-R	CAATAAACAAGTTAGATCTTTACTTATCGTCGTCATCCT
CMV-p72-F	ATATAAGCAGAGCTGCTAGCGCCACCATGTACCCATACGATGTTCCAGA
CMV-p72-R	TGCCCTCTCCACTGCCGCATGCAGCGTAATCTGGAACATCGTATG
CMV-CD2v-F	ACGTCGAGCACGCGCATGCGACATTGATTATTGACTAG
CMV-CD2v-R	ACTTCCTCTGCCCTCTCCACTGCCAGATCTCTTATC
CMV-pp62-F	GCATTGATTATTGACTAGTTATTAATAGTAATCAATTA
CMV-pp62-R	ATAAACAAGTTGCGGCCGCTGGCATGCTAGCTTAAGCGT
p54-30a-F	TCGAGTGCGGCCGCAAGCTTCAGGCTGTTCTCCAGGTCC
p54-30a-R	CTGATATCGGATCCGAATTCAGCCGCAAGAAGAAGGCCG
p72-30a-F	TCGAGTGCGGCCGCAAGCTTGGTGCTGTACCGCAGCACG
p72-30a-R	CTGATATCGGATCCGAATTCCCCGAGATCCACAACCTGT
CD2v-30a-F	TCGAGTGCGGCCGCAAGCTTGTTGGAGGACAGGGTCAGG
CD2v-30a-R	CTGATATCGGATCCGAATTCGTGAGCTTCAACAAGACCA
pCold-pp62-F	CGGTACCCTCGAGGGATCCATGCCCTCCAACATGAAGCA
pCold-pp62-R	ATCTAGACTGCAGGTCGACGTCGAAGTCGATCAGGGGCAG
sgRNA1/2(UL24/TK)-1F	CACCGGCCTCGCCGAGTACGTCGC
sgRNA1/2(UL24/TK)-1R	AAACGCGACGTACTCGGCGAGGCC
sgRNA1/2(UL24/TK)-2F	CACCGCGCCTTCACGTCGGAGATG
sgRNA1/2(UL24/TK)-2R	AAACCATCTCCGACGTGAAGGCGC
sgRNA3/4(UL24/TK)-3F	CACCGCATCGACGCCGGTACTGCGG
sgRNA3/4(UL24/TK)-3R	AAACCCGCAGTACCGGCGTCGATGC
sgRNA3/4 (UL24/TK)-4F	CACCGAGAAACCGGAAGTGACGAA
sgRNA3/4 (UL24/TK)-4R	AAACTTCGTCACTTCCGGTTTCTC
Detect-p54-F	CCACTTCACAAGTCGGAGGCTTA
Detect-p54-R	CCAGTTTGGAAGCATCCATCATTTC
Detect-p72-F	GCTCTTACTGACTGGCATGAG
Detect-p72-R	CGCAGCTCTAGGAGCATGTG
Detect-CD2v-F	CTCTTCTTGGATATCTGGAGGAACTGG
Detect-CD2v-R	AATGACGCTTATGTTGTTGCTGATGG
Detect-pp62-F	TTGGGAGGGTGAGGGACT
Detect-pp62-R	GAACGGTGAAGGCGACAG
TK-F	CGCACTCTGTTCGACACGGA
TK-R	GCTGATGTCCCCGACGATGA
UL24-F	GCCGCCTTATCATCCCCGCT
UL24-R	GAGCTCAAGACGTGCCGCTT
gI/gE-F	AGCCCAAGATGACGTTGGCC
gI/gE -R	CTCGGACACGTTCACCAGAT
EGFP-F	AGCTGACCCTGAAGTTCATC
EGFP-R	AACTCCAGCAGGACCATGTG
mCherry-F	ATCATCAAGGAGTTCATGCG
mCherry -R	AGGTGATGTCCAACTTGATG

## Data Availability

The data that support the findings of this study are available from the corresponding author upon reasonable request.

## Supporting Information

Supporting Information Figure S1. Expression, purification, and identification of ASFV p54, p72, CD2v, and pp62 proteins, and establishment of ELISA method.
