# Supplementary material for: Construction and Evaluation of Quadruple-Gene-Deleted Pseudorabies Virus Platforms for ASFV Antigen Delivery
Source: Transbound Emerg Dis. 2025 Jul 16;2025:3628600. doi: 10.1155/tbed/3628600 (PMC12286666; doi:10.1155/tbed/3628600)
Supplement: Supporting Information — Figure S1. Expression, purification, and identification of ASFV p54, p72, CD2v, and pp62 proteins, and establishment of ELISA method. [file 3628600.f1.docx]

**Supplementary information**


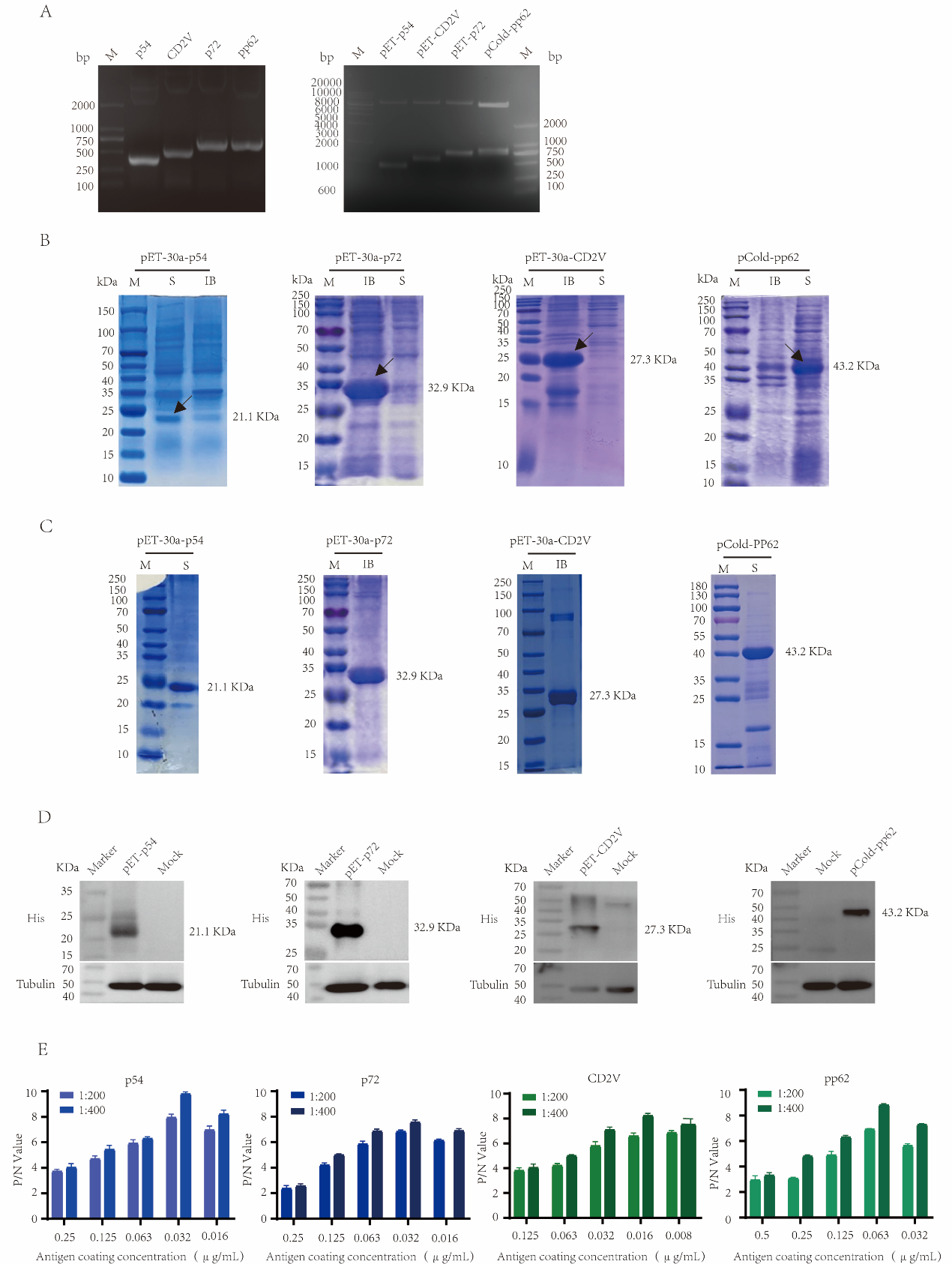


**Figure S1. Expression, purification and identification of ASFV p54, p72, CD2v, and pp62 proteins, and establishment of ELISA method.** (A) PCR amplification and restriction enzyme digestion identification of p54, p72, CD2v, and pp62 gene fragments. (B) SDS-PAGE analysis of p54, p72, CD2v, and pp62 recombinant proteins after IPTG induction, arrows indicate the target protein bands. (C) Purification of His-tagged recombinant proteins by Ni-NTA affinity chromatography (D) Western blot analysis of purified proteins using anti-His antibody (1:1000 dilution). (E) Optimization of indirect ELISA method: using P/N ratio (positive/negative ratio) as evaluation criterion, checkerboard titration was performed to determine the optimal coating antigen concentration and serum dilution, to achieve maximum detection specificity and sensitivity.
